# Metformin promotes susceptibility to experimental *Leishmania braziliensis* infection

**DOI:** 10.1590/0074-02760200272

**Published:** 2020-11-16

**Authors:** Filipe Rocha Lima, Lais de Melo Ferreira, Tainá Alves Malta, Icaro Bonyek-Silva, Reinan Lima Santos, Natália Machado Tavares, Edgar Marcelino de Carvalho Filho, Sérgio Arruda

**Affiliations:** 1 Fundação Oswaldo Cruz-Fiocruz, Instituto Gonçalo Moniz, Laboratório Avançado de Saúde Pública, Salvador, BA, Brasil; 2 Fundação Oswaldo Cruz-Fiocruz, Instituto Gonçalo Moniz, Laboratório de Interação Parasito-Hospedeiro e Epidemiologia, Salvador, BA, Brasil; 3 Fundação Oswaldo Cruz-Fiocruz, Instituto Gonçalo Moniz, Laboratório de Pesquisa Clínica, Salvador, BA, Brasil; 4 Universidade Federal da Bahia, Salvador, BA, Brasil; 5 Universidade Estadual da Bahia, Departamento de Ciências da Vida, Salvador, BA, Brasil

**Keywords:** metformin, cutaneous leishmaniasis, immunomodulation, susceptibility, infection

## Abstract

**BACKGROUND:**

Metformin (MET) is a hypoglycemic drug used for the treatment of diabetes, despite interference in host immunity against microorganisms. Cutaneous infection caused by pathogens such as *Leishmania braziliensis* (*Lb*), the agent responsible for cutaneous leishmaniasis (CL) in Brazil, represents an interesting model in which to evaluate the effects associated with MET.

**OBJECTIVE:**

To evaluate the modulatory effect of MET in *Lb* infection.

**MATERIAL AND METHODS:**

Experimental study of *Lb* infection and MET treatment in BALB/c mice and Raw 264.7 macrophages.

**FINDINGS:**

MET treatment interfered with lesion kinetics, increased parasite load and reduced macrophage proliferation. Low concentrations of MET in *Lb* culture allow for the maintenance of stationary parasite growth phase. *Lb*-infected cells treated with MET exhibited increased parasite load. While both MET and *Lb* infection alone promoted the production of intracellular reactive oxygen species (ROS), reduced levels of ROS were seen in MET-treated *Lb*-infected macrophages.

**MAIN CONCLUSION:**

Experimental treatment with MET interfered with the kinetics of cutaneous ulceration, increased *Lb* parasite load, altered ROS production and modulated cellular proliferation. Our experimental results indicate that MET interfere with the evolution of CL.

Cutaneous leishmaniasis (CL) is a clinical form of the anthropozoonotic disease complex caused by protozoa of the genus *Leishmania*.^1^ In Brazil, CL is commonly caused by *Leishmania braziliensis* (*Lb*) and is clinically characterised by one or more oval-shaped ulcerative lesions, or rounded, granulomatous-bottomed ulcerations with well-defined raised borders.^2^ ^,^ ^3^ The low parasitic burden found in lesions results from a Th1-mediated intense inflammatory response, in addition to cytokine production, such as IFN-γ, TNF, IL-12 and IL-2. Intracellular death in these parasites results from the microbicidal action of IFN-γ-stimulated macrophages. However, exacerbated inflammation provokes tissue damage and initiates a pathological process culminating in the formation of ulcers on the skin of the host.^3^ ^,^ ^4^ ^,^ ^5^

In Brazil, pentavalent antimonials (Sb^+5^) are the first-line drugs for *Lb* infection treatment. Despite high efficacy, the numerous side effects, such as cardiac, renal and hepatic toxicity, induced by these drugs consequently restricts use to certain groups of patients.^2^ In addition, recent studies have demonstrated increasing resistance to these antimonials in several Latin American countries, as well as in India.^6^ ^,^ ^7^ ^,^ ^8^ In light of this scenario, several studies have investigated alternative treatment strategies, such as combined therapy involving Sb^+5^ and immunomodulators, such as pentoxifylline, an inhibitor of TNF production, which seemed to accelerate cure in patients.^1^ ^,^ ^9^

The immunomodulatory effects of antidiabetic drugs, such as metformin (MET), has been described in cancer and infectious diseases, including tuberculosis and pneumonia caused by *Legionella pneumophila*.^10^ ^,^ ^11^ Antidiabetic drug-induced effects include 5’ AMP-activated protein kinase (AMPK) activation, signal transducer and activator of transcription 3 (STAT3) inhibition, and the increased production of mitochondrial reactive oxygen species (ROS).^12^ ^,^ ^13^ Although described in other contexts, little is known about the action of MET in parasitic infections, such as *Lb* Considering the high prevalence of CL in Brazil, together with this drug’s ample availability and low cost, the present study aimed to analyse the effects of MET on the immune response against *Lb* in an experimental mouse model.

## MATERIALS AND METHODS

*Experimental model design* - After receiving ethical approval by the local Institutional Review Board for Animal Experimentation (protocol 013/2017), 6-week-old male isogenic BALB/c mice were obtained from the Animal Care Facility of the Instituto Gonçalo Moniz, Fundação Oswaldo Cruz (IGM-Fiocruz) in Salvador, Bahia, and maintained under controlled conditions (temperature 22 ± 2ºC, 50% humidity, 12-hour light/dark cycles). Mice were divided into three groups of five according to MET treatment and *Lb* infection status. Group 1: *Lb* infection; Group 2: MET treatment alone; Group 3: *Lb* infection and MET treatment.

*Metformin hydrochloride treatment* - Mice from Groups 2 and 3 received continuous pre-treatment for six weeks (prior to infection procedures) with an oral solution containing metformin hydrochloride (Hcl) (Galena Biopharma, India) at a dosage of 500 mg/kg diluted in water. Following *Lb* infection procedures, mice in Groups 2 and 3 continued to receive MET solution orally for an additional six weeks.

*Leishmania braziliensis infection* - Groups 1 and 3 were intradermally infected in the left ear using a 30G needle syringe containing 10 μL of 2 x 10^5^ stationary-phase metacyclic *Lb* promastigotes (strain MHOM/BR/01/BA788). Parasites were cultivated in Schneider’s medium (Sigma Chemical Co., St Louis, MO, USA), supplemented with 20% inactivated foetal bovine serum (FBS) (Gibco, USA), L-glutamine (2 mM), penicillin (100U / mL) and streptomycin (100 mg / mL), for 5-7 days under 23ºC until reaching stationary phase.

The diameter of the infected ear was measured weekly in Groups 1 and 3 with the aid of a digital caliper. Measurements were expressed in millimeters (mm) and used to compare between groups.

*Parasite load quantification -* Parasitic load was determined in Groups 1 and 3 based on a limiting dilution assay^14^ at six weeks after infection, when the animals were euthanised and their ipsilateral lymph nodes and infected ears were removed. Following extraction under sterile conditions, the obtained mouse organs were macerated using a 40 µm pore size cell strainer, with the addition of 2 mL Roswell Park Memorial Institute (RPMI) medium supplemented with 10% FBS, L-glutamine (2 mM), penicillin (100 U / mL) and streptomycin (100 mg / mL). Collected cells were then centrifuged at 1500 rpm under 4ºC for 10 min. The precipitate was resuspended in 1 mL of supplemented Schneider medium; 20 μL aliquots were serially diluted on a 96-well flat-bottomed plate containing 180 μL/well of supplemented Schneider medium. Eight serial dilutions were performed in triplicate. Plates were sealed and incubated in a Biochemical Oxygen Demand (BOD) incubator at 23ºC for 10 days. Wells were observed under an inverted optical microscope (Nikon) at 20× magnification, with daily readings taken beginning on the third day of cultivation to register viable promastigotes.

*Cell viability testing* - Raw 264.7 macrophages were plated at a concentration of 10^5^ cells/well on a CELLSTAR^®^ 96-well flat plate containing 200 μL/well of Dulbecco’s Modified Eagle’s medium (DMEM) (GibcoTM) supplemented with 10% FBS (Gibco), L-glutamine (2 mM), penicillin (100U/mL) and streptomycin (100 mg/mL). Experiments were performed in triplicate under the following conditions: positive control (viable cells in DMEM medium), negative control [cells grown in 1x phosphate-buffered saline (PBS)], R white control (DMEM medium) and macrophages incubated with 2, 2.5, 5 and 10 mM of MET hydrochloride for 24 h at 37ºC under 5% CO_2_. Next, 20 μL from each well was removed, followed by the addition of 20 μL of Alamarblue^TM^ Cell Viability Reagent (ThermoFisher Scientific), followed by a 4-hour reincubation period under the conditions described above in the absence of light. Readings were performed on a spectrophotometer at wavelengths of 570 nm and 600 nm, with results interpreted following the manufacturer’s protocol. The selected concentration for subsequent experimentation was determined to be 2 mM of MET.

*Macrophage viability assays* - Cell growth kinetics were investigated using a 24-well plate assay (CELLSTAR^®^) performed in triplicate with 10^5^ macrophages/well under the following conditions: 1) no MET; 2) treatment with 2 mM MET, followed by incubation; 3) cells pre-treated with 2 mM of MET six days prior to receiving an additional 2 mM MET, followed by incubation. Counts were performed at 24, 48, 72 and 96 h of cultivation at 37ºC under 5% CO_2_ in supplemented DMEM (1 mL) (GibcoTM). At each cell counting timepoint, DMEM medium was discarded and cells were removed by enzymatic action using 1 mL 0.25% trypsin-EDTA (Sigma-Aldrich) for 1 min at 37ºC under mechanical homogenisation with a micropipette. The aspirated volume (1 mL) was inactivated with 9 mL supplemented DMEM and centrifuged at 1200 rpm under 4ºC for 10 min. The medium was discarded and obtained cellular pellet was resuspended in 1 mL of supplemented DMEM. Trypan Blue (Sigma-Aldrich) was added to the cell suspension aliquots at a ratio of 1:10. Cell viability was then assessed by counting in a Neubauer chamber at 400x magnification.

*Influence of metformin on L. braziliensis growth* - Metacyclic *Lb* promastigotes (5 x 10^5^/mL) were placed in 25 cm^2^ cell culture flasks (CELLSTAR®) containing 5 mL of supplemented Schneider’s medium and grown in a BOD at 23ºC. Cultivation was performed in duplicate under the following conditions: 0, 2, 5 or 10 mM of MET. Promastigote growth was measured via daily counts for six days by removing 10 μL from culture flasks, which was placed in a Neubauer chamber and observed under an E200 (Nikon) optical microscope at 400x magnification.

*Raw 264.7 macrophage infection with L. braziliensis* - Macrophages were plated at a concentration of 10^5^ cells/well on 24-well plates (CELLSTAR^®^) in 300 μL of supplemented DMEM medium, followed by the addition of circular coverslips (13 mm) for macrophage adhesion.

Cells were cultivated under the following conditions: 1) negative control without treatment or infection; 2) positive control with 100 ng/mL lipopolysaccharide (LPS) treatment; 3) 2 mM MET treatment without infection; 4) infection with *Lb*; 5) infection with *Lb* and 2 mM MET treatment; 6) 100 ng/mL LPS and 2 mM MET treatment; 7) infection with *Lb*, 100 ng/mL LPS and 2 mM MET treatment. Cells under conditions 3, 5, 6 and 7 were pretreated with 2 mM MET for five days prior to challenge, and received an additional 2 mM MET following infection procedures. The multiplicity of infection (MOI) used was 10:1 parasites/macrophage, with a 4-hour incubation at 37ºC under 5% CO_2_. Experimental conditions were performed in quadruplicate and the experiment was repeated twice.

*Parasite infection rate and quantification* - Coverslips were washed thrice with 1 mL PBS 1x and then subjected to rapid panoptic staining (Laborclin). Rapid panotic number 1 (300 μL) was added to the wells and stored for 10 min in a refrigerator at 2 to 8ºC, followed by 300 μL of panoptic number 2 for 1 minute at RT, 300 μL of panoptic number 3 for 10 seconds at RT and finally washed thrice with 1 mL of distilled water with the aid of a 1000 μL micropipette during the entire process. The coverslips were then mounted using Entellan (Sigma-Aldrich) on 26x76 mm glass slides for further evaluation under an optical microscope (Nikon) at 100x magnification. The number of infected cells (presence of parasites in intracellular medium) was totaled out of 100 counted cells, with values expressed as percentages, and the total number of parasites was counted inside infected cells.

*Evaluation of intracellular parasite viability* - After 4 h of infection, the wells were washed thrice with 300 μL 0.9% NaCl solution at 37ºC to remove any non-internalised promastigotes. Prior to washing, 200 μL/well of supplemented Schneider medium was added and the sealed plate was incubated in a BOD at 23ºC for 96 h. The number of promastigotes in 10 μL aliquots were counted in a Neubauer chamber at 400x magnification.

*ROS quantification in culture supernatant* - Culture supernatant was distributed on two 96-well flat-bottomed plates (Greiner Bio-One) in triplicate. To measure ROS in the supernatant, an alternative methodology involving Griess reaction (Sigma-Aldrich) was used. The Griess method allows, via colorimetric reaction, the measurement of nitric oxide (NO) production through nitrite conversion using spectrophotometry. Two experimental conditions were used: wells in one plate received the addition of hydroxylamine (NH_2_OH), while another did not. Optical density (OD) values were transformed in concentrations (μM) by measuring differences in OD readings between the two plates, thus allowing for an indirect evaluation of ROS in the culture supernatant, due to the inhibition of the nitrification reaction via the presence of the NH_2_OH intermediate substrate.

*Quantification of intracellular ROS* - Cells were submitted to a CellROX Oxidative Stress Reagent assay (ThermoFisher Scientific). All wells containing macrophages were washed thrice with 1x PBS, and 300 μL/well of supplemented DMEM medium and CellROX reagent were added. After being incubated in the dark at 37ºC under 5% CO_2_ for 30 min, wells were washed three times again with 1x PBS, and the cell coverslips were fixed with 3.7% paraformaldehyde (PFA) for 15 min, then mounted on a 23x76 mm glass slide with 5 μL of 4‘, 6-diamino-2- phenyl indole (DAPI) in antifade mounting medium (ThermoFisher Scientific). Subsequently, the slides were analysed under an inverted fluorescence microscope (Leica) in the range of 485/520 nm, and images of fields containing eight cells each were captured to quantify the corrected total fluorescence coefficient (CTCF) using OlyVIA 7.0 software (Olympus). This analytic strategy consists of measuring fluorescence in the area occupied by a cell, averaging values from the eight cells analysed, and then subtracting the fluorescence background average from five extracellular spaces.

*Statistical analysis* - The D’Agostino test was used to classify the distribution of samples as parametric or nonparametric. The Mann-Witnney or Kruskal-Wallis tests were used for nonparametric samples, while the t-test or one way analysis of variance (ANOVA) was employed for parametric distribution. The Chi-square test for trend was also used. Analyses were performed using GraphPad Prism v.7 software (GraphPad Software®, San Diego, CA), and p values less than or equal to 0.05 were considered significant.

## RESULTS

**Fig. 1:**
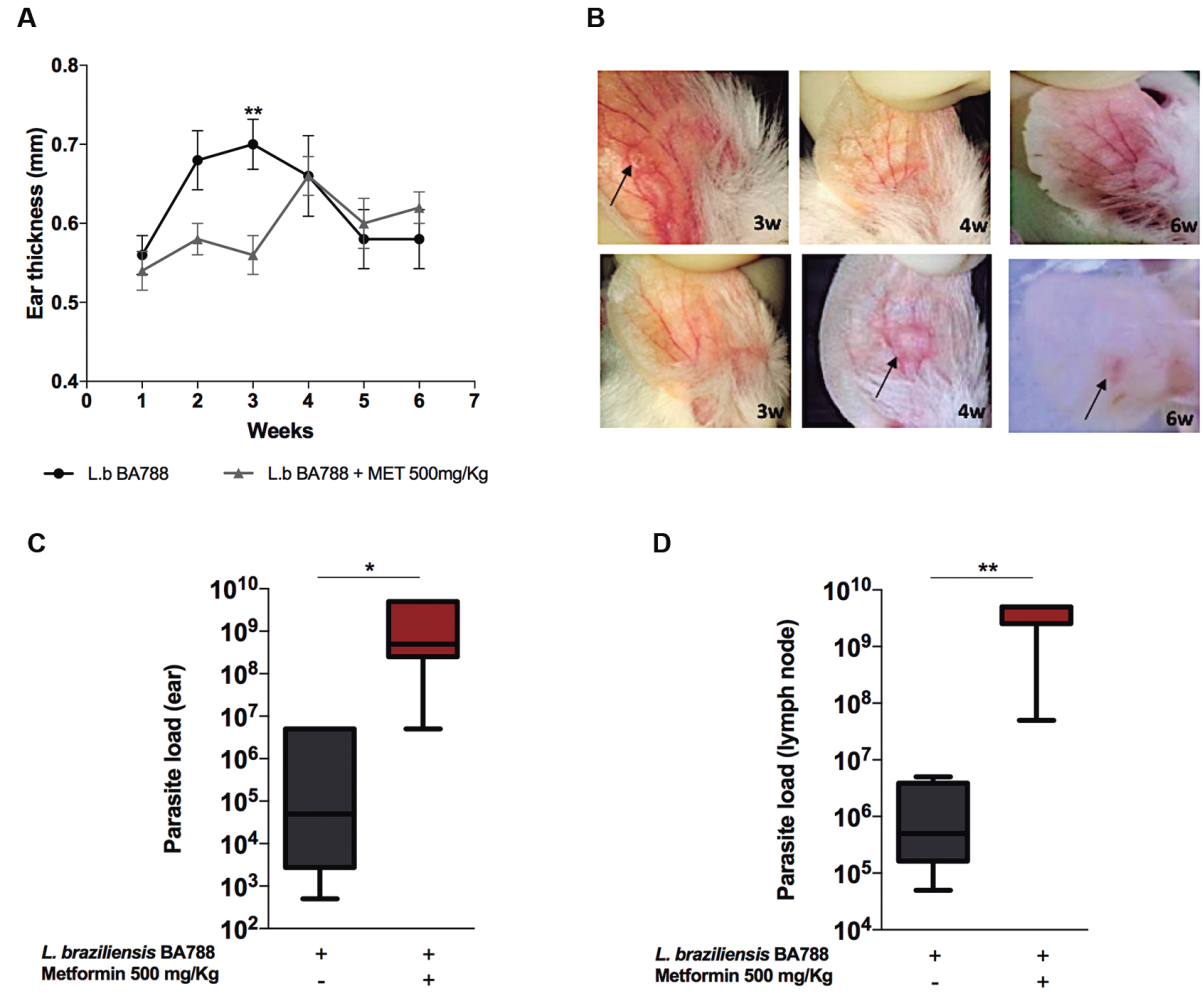
monitoring of skin lesion appearance and parasite load in an experimental model of infection with *Leishmania braziliensis* (*Lb*). Metformin (MET)-treated mice received the first dose (500 mg/kg) six weeks before infection, and continued treatment at the same dosage for six additional weeks after infection. The untreated group had no contact with MET at any time during experimentation. Graph of lesion size in infected animals measured at the site of infection, the left ear, with the aid of a digital caliper (A). Photographic documentation of the left ear of the group infected with *Lb* and not treated with MET (upper panels) and mice infected with *Lb* and treated with MET (lower panels), during the third (3w), fourth (4w) and sixth weeks (6w) post-infection (B). Comparisons of parasite load between groups infected with *Lb* and not treated with MET versus *Lb-*infected and MET-treated in the left ear (C) and lymph node (D) six weeks post-infection, measured by limiting dilution assay. Statistical analysis performed using one-way ANOVA (A) and Mann-Whitney test (C-D). Asterisks indicate significant differences, respectively (**p = 0.0048; *p = 0.02; **p = 0.01).

*MET delays the appearance of Leishmania papules and modifies lesion size* - Three weeks after inoculating metacyclic promastigotes in stationary phase, G1 (*Lb*) animals presented a 20% larger ear thickness compared to G3 (*Lb* + MET): mean 0.7 mm (± 0.07; p = 0.0048), small ulcerated papule with acneiform aspect and cutaneous hyperemia. On the other hand, lesion size in G3 was 3.4% smaller than G2 (control) of 0.56 mm (± 0.055), ie, showing no evident tissue damage. In the 4th week of infection, lesion size in G1 decreased by 5.7%, while group G3 increased by 15.1%, demonstrating the appearance of acneiform papules without ulceration. At six weeks after infection, the absence of ulcers was seen in non-MET (G1) animals, with ear thickness averaging 0.58 mm (± 0.084), just 0.8% different from G2, the uninfected group (Fig. 1A-B).

*MET promotes increased parasitic load in lesions and ipsilateral lymph node* - Six weeks after infection, all animals were euthanised and the left ears and cervical lymph nodes were resected in G1 (n = 05) and G3 (n = 05). Mouse organs were macerated and then cultivated in serial dilutions for parasitic load measurement. At the site of infection (Fig. 1C) and in the cervical lymph nodes (Fig. 1D), G3 presented the highest limiting dilution positivity (5 x 10^6^ parasites/mL), 10^3^ more promastigotes compared to G1 (p = 0.02; p = 0.01, respectively).

*MET exerts no cytotoxic effects and inhibits Raw 264.7 macrophage growth* - Cell viability following exposure to different MET concentrations was evaluated in cultures of Raw 264.7 macrophages, with an absence of any observable cytotoxic effects arising due to MET within 24 h. Cell viability values ranged from 85 to 100% relative to the positive control, i.e. cells that did not receive MET in supplemented DMEM medium.

**Fig. 2:**
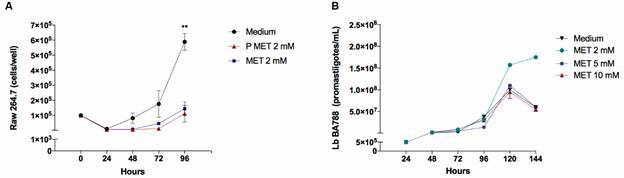
cultivation of Raw 264.7 macrophages and *Leishmania braziliensis* (*Lb*) promastigotes exposed to metformin (MET). Cell growth kinetics investigated in triplicate assays (10^5^ cells/well) under the following conditions: no MET (Medium); treatment with 2 mM MET; cells pre-treated with 2 mM MET continuously for six days prior to receiving an additional 2 mM MET. Counts performed at 24, 48, 72 and 96 h after cultivation (A). Metacyclic *Lb* promastigote (5 x 10^5^/mL) growth measured daily at 24, 48, 72, 96, 120, 144 h under optical microscopy at 400x magnification, in response to the following conditions: 0, 2, 5 or 10 mM of MET (B). Statistical analysis performed using one-way ANOVA (A). Asterisks indicate significant differences (Medium vs 2 mM MET; p = 0.0024) and (Medium vs 2 mM P MET; p = 0.0016).

Using the selected concentration for subsequent experimentation was determined to be 2 mM of MET, a cell viability assay (1 x 10^5^ cells/well) was performed to quantify the number of viable cells after 24, 48, 72 and 96 h of MET exposure by comparing cells pretreated for six days in MET and those without previous drug exposure. After 96 h, cultured cells without prior exposure to MET achieved an average growth of 1.4 x 10^5^ cells, i.e. 22.5% higher than pretreated cells. Cultures without MET yielded an average of 5.9 x 10^5^ cells/well after 96 h, representing a 76.3% higher proliferation rate compared to macrophages not previously exposed to MET (p = 0.0024), versus 81.3% more compared to previously treated cells (p = 0.0016) (Fig. 2A).

*Low MET concentrations increase Lb promastigote growth* - After verifying that MET interferes with macrophage growth *in vitro*, we verified the effect of this drug on *Lb* promastigote cultivation *in vitro* by growing 5 x 10^5^ metacyclic promastigotes/mL in 5 mL of DMEM. Cultivation was quantified daily for six days until proliferation was observed to decrease. On the 6th day of culture, 34.3% higher growth was seen in cells receiving 2 mM MET compared to those that did not receive the drug, with an average of 1.7 x 10^8^ parasites/mL and demonstrated continuance of in log phase growth. Under culture conditions involving 5 mM MET, no difference was seen compared to drug-free culture (average of 6 x 10^7^ parasites/mL). Using 10 mM MET, 8.5% less proliferation was observed compared to drug-free culture (average of 5.5 x 10^7^ parasites/mL) (Fig. 2B).

**Fig. 3:**
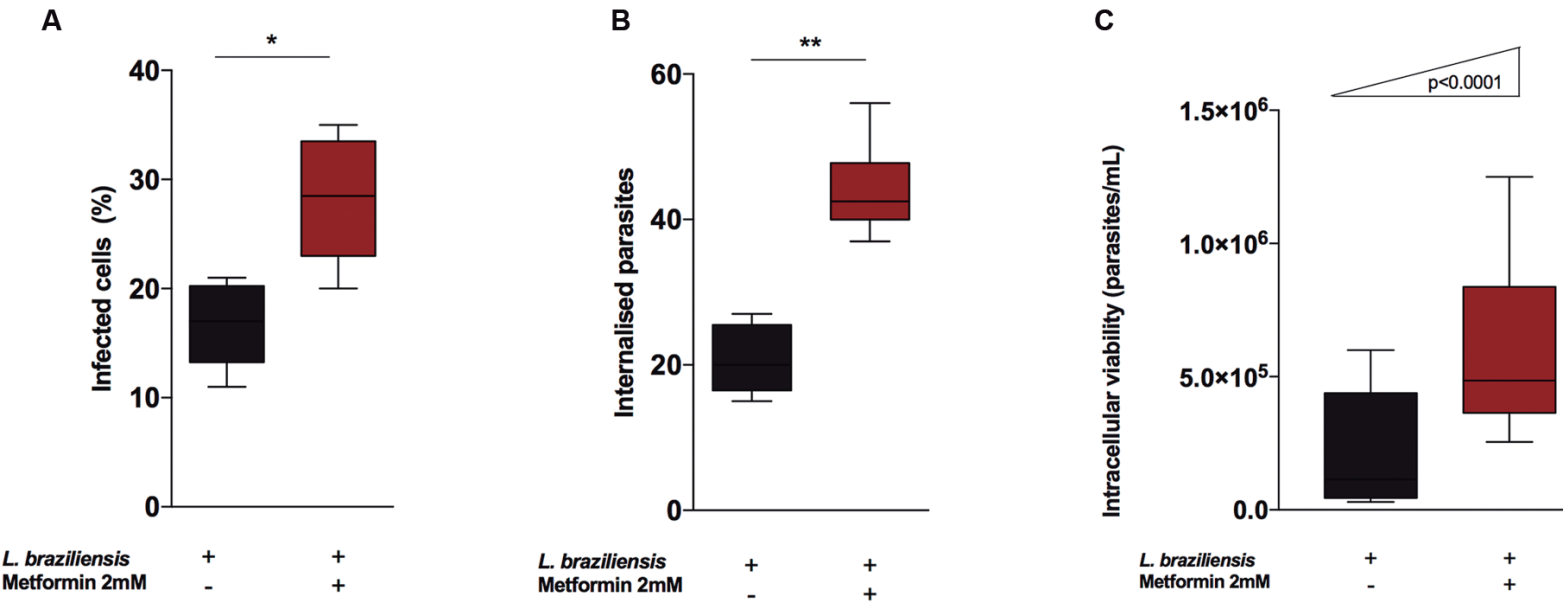
comparative evaluation of parasitic load and intracellular viability of *Leishmania braziliensis* (*Lb*) in Metformin (MET)-treated and untreated Raw 264.7 macrophages (10^5^ cells/well). The MET group was previously treated with 2 mM MET for six days before challenge and received an additional 2 mM after infection (MOI 10:1 parasites/macrophage). Graphical representation of infection rate in 100 cells, with results expressed as percentages (A), quantification of internalised parasites in infected cells by optical microscopy at 1000x magnification (B). Intracellular parasitic viability was evaluated after 96 h and promastigotes were counted in infected cells (C). Statistical analysis was performed using Mann-Whitney test (A-B) and Chi-square for trend test (C). Asterisks indicate significant differences (*p = 0.006; **p = 0.02).

*In vitro MET treatment resulted in increased infection rate and parasitic load in Raw macrophages 264.7* - After a 4-hour infection period, the percentage of infected cells (Fig. 3A) and the number of parasites relative to the quantified cells (Fig. 3B) were counted. The addition of MET to cell cultures was found to interfere with the frequency of infected macrophages (p = 0.006) compared to untreated cells. The number of parasites/cells was 46.8% higher in infected cells treated with MET compared to untreated cells (p = 0.002).

Similar to our results regarding infection rate and parasites counts, intracellular parasitic viability analysis revealed an increase in parasite viability at 96 h in macrophages treated with MET compared untreated cells (p < 0.0001) (Fig. 3C).

Taken together, these data suggest that MET-treated cells may decrease the microbicidal potential of macrophages during *Lb* infection.

**Fig. 4:**
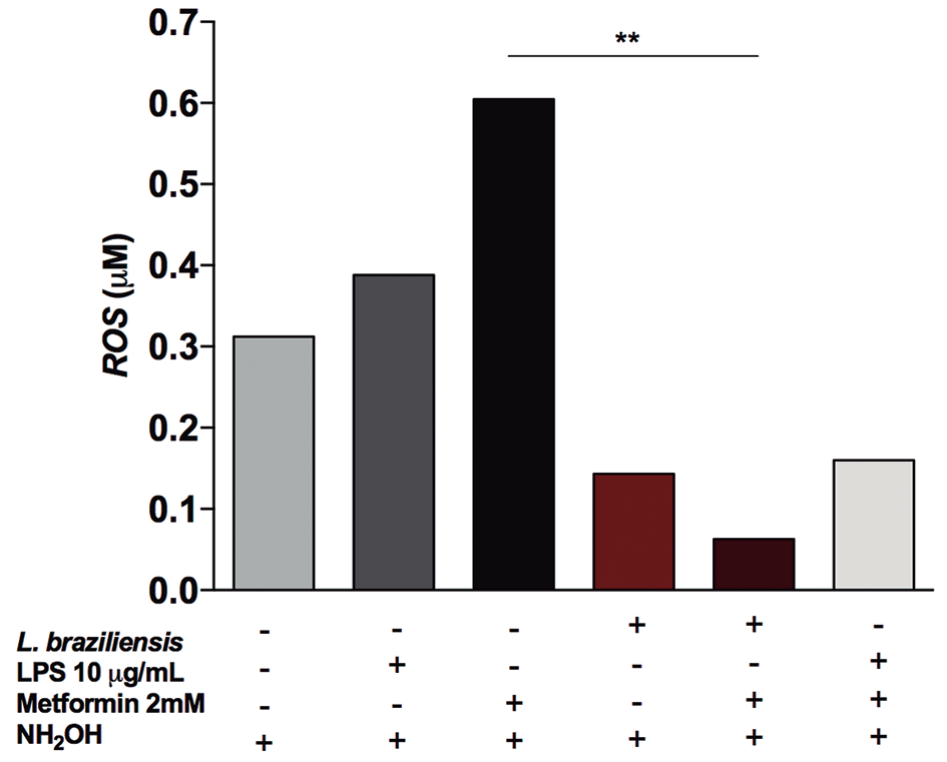
production of reactive oxygen species (ROS) measured in the culture supernatant of infected Raw 264.7 macrophages (10^5^cells/well, multiplicity of infection (MOI) 10:1 parasites/macrophage) using an alternative methodology involving the Griess reaction. Optical density values were transformed into concentrations (μM). Statistical analysis was performed using Kruskal-Wallis test. Asterisks indicate significant differences (**p = 0.007). LPS: lipopolysaccharide. NH2OH: hydroxylamine.

**Fig. 5:**
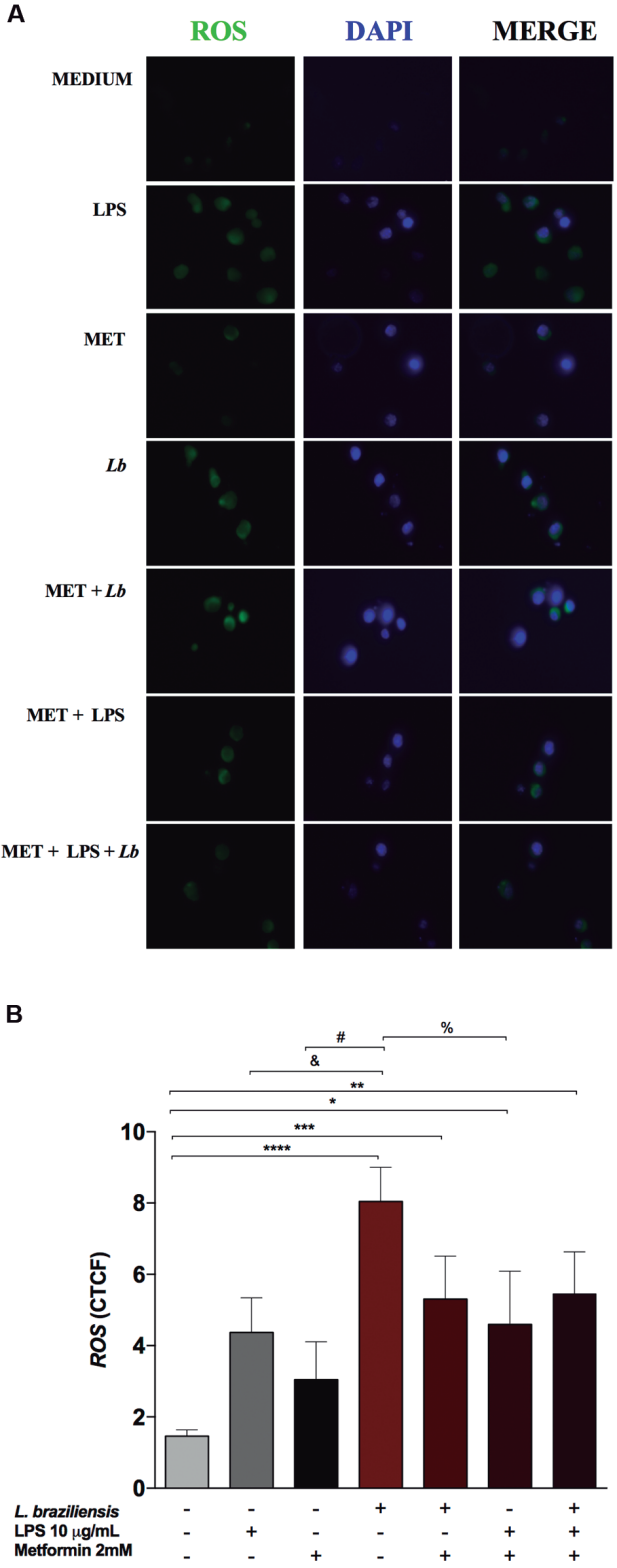
intracellular quantification of reactive oxygen species (ROS) in Raw 264.7 macrophages (10^5^ cells) infected with *Leishmania braziliensis* (*Lb*) multiplicity of infection (MOI) 10:1 parasites/macrophage under different conditions. Representative images of cell labeling by immunofluorescence (A) and quantification of corrected total fluorescence coefficient (B). Statistical analysis performed using the one-way ANOVA. Symbols indicate significant differences (**** p = 0.0001; *** p = 0.008; * p = 0.03; ** p = 0.006; & p = 0.01; #p = 0.0008; % p = 0.01). LPS: lipopolysaccharide.

*MET suppresses the release of ROS in the extracellular environment and intracellular ROS production in Lb-infected Raw 264.7 macrophages* - ROS from macrophages was quantified in culture supernatants. After infection with *Lb*, MET treatment suppressed ROS production by 89.6% compared to uninfected treated cells (p = 0.007) (Fig. 4). In Fig. 5, representative microscopic fields illustrating corrected total cell fluorescence intensity allowed for the qualitative verification of fluorescence with respect to ROS (A), DAPI (B) and overlap (C) (Fig. 5A). Our results indicate a significant increase in ROS production in *Lb-*infected cells, evidencing the effect of *Lb* on macrophage activation compared to uninfected cells (p < 0.0001). *Lb* was found to induce a superior effect compared to stimulation with LPS (p = 0.01) or MET (p = 0.0008), and also resulted in higher ROS production compared to LPS-stimulated cells and treated with MET (p = 0.01). This analysis highlights differences in the production of reactive mediators compared to the negative control, under stimulation with MET treatment and *Lb* infection (p = 0.008), MET and LPS (p = 0.03) or MET, LPS and *Lb* (p = 0.006). Despite achieving statistical significance (p = 0.08), we also found that MET reduced intracellular ROS production by 34.1% in *Lb* infection compared untreated infected macrophages (Fig. 5B).

## DISCUSSION

The present results demonstrate that MET treatment in *Lb*-infected mice delays the appearance of papules characteristic of *Lb* infection; however, this drug not found to prevent ulceration. A study by Qing^15^ highlights the molecular mechanisms of MET in inducing the AMPK / mTOR axis by promoting NLRP3 inhibition as a crucial event in M2 macrophage induction, as well as promoting rapid tissue healing. Another study by Ochoa-Gonzalez^16^ stated that MET blocks mTOR action, shortens the cell cycle, reduces *in vitro* keratinocyte cell proliferation, negatively impacted healing in an experimental model and worsened the clinical condition of foot ulcers in diabetic patients, suggesting that MET interferes with tissue remodeling, lesion size and lesion healing time.

MET induces AMPK activation by modulating the mitochondrial ADP / ATP balance, resulting in the production of mROS in macrophages, which increases the microbicidal potential of these cells in the context of other pathogenic infections, such as *Mycobacterium tuberculosis* ^17^ and *L. pneumophila*.^12^ Other studies have presented evidence that by interfering with mitochondrial metabolism and adaptive immunity in *Plasmodium* infections, MET could exert a protective effect in malaria.^18^ ^,^ ^19^ The use of MET treatment in the experimental murine *Lb* infection model employed herein resulted in a 1,000-fold increase in the number of parasites at the inoculation site and draining lymph nodes compared to untreated mice. These findings suggest that MET exacerbates the production of ROS, a marker of cellular oxidative stress, and results in increased susceptibility to *Lb* Novais et al.^20^ reported that although *Leishmania* parasites are sensitive to ROS, the respiratory explosion that occurs in macrophages that are not activated by adaptive immunity in response to infection is insufficient to enable parasite killing. Fukai et al.^21^ stated that some parasite species may induce antioxidant responses as an escape mechanism. Khouri et al.^22^ found significant increases in the expression and activity of superoxide desmutase-1 (SOD-1) occurring in human macrophages infected with *L. amazonensis*, which directly favors parasite survival.

The pleiotropic effects of MET are also known to attenuate monocyte differentiation in macrophages, as well as inflammation-inducing events.^14^ We found that 2 mM MET inhibited the ability of macrophages to proliferate independent of the time of drug exposure, and did not interfere with cell viability. With regard to *Lb* growth, treatment with 2 mM MET did not affect parasite growth, which remained in stationary growth phase, however this was not the case when using higher doses or the absence of MET. The Raw 264.7 macrophage cell line presents a doubling time of 16-24 h.^23^ Accordingly, since MET did not modulate cell proliferation in 4-hour infection assays, this suggests that increased parasitic load and viability observed herein may be influenced by exposure to MET.

Wang et al.^24^ demonstrated the effects of MET in cancer, detailing that this drug inhibits abnormal cell proliferation by modifying the tumor microenvironment, which causes decreased angiogenesis, as well as by altering the tumor-associated macrophage phenotype that subsidises tumor development.^24^ In hepatocellular carcinoma, Del Campo et al.^25^ and Choi and Roberts^26^ corroborated findings reaffirming the direct action of MET in AMPK induction, mTOR inhibition, cell cycle suppression and the consequent proliferation of tumor cells.

Following our evaluations of the effect of MET in isolated macrophage cultures, MET was investigated in *Lb* infection *in vitro*. In addition to increased numbers of infected cells and parasitic load, in intracellular parasite viability was found to increase by 36% in MET treated macrophages within 96 h of infection compared to untreated infected cells. Considering that one of the main mechanisms of pathogen intracellular death is the production of ROS,^20^ and that MET interferes with the production of Mros,^12^ ^,^ ^17^ we hypothesise that, during the course of infection, *Lb* interferes with MET-induced ROS levels, which promotes increased parasite viability. In macrophage culture supernatants, we found that MET increases the release of ROS in uninfected cells; however, cells infected with *Lb* exhibited a significant decrease in ROS production following treatment with the drug*.*

The cultivation of uninfected macrophages with MET resulted in a 48.1% increase in mean intracellular ROS production, while stimulation by *Lb* infection promoted a 37.8% greater synthesis compared to MET. Moreover, the introduction of MET in infected macrophage cultures was found to decrease intracellular ROS production by 34%. Quintela-Carvalho et al.^27^ described that, in excess, ROS can be highly harmful to parasites and host cells. Therefore, to circumvent oxidative stress, host cells produce antioxidants, such as SOD-1, which catalyse the dismutation of superoxide in oxygen and hydrogen peroxide.^21^ The mechanisms elucidated in the literature indicate that some parasites may induce antioxidant responses as an escape mechanism. In human macrophages, *L. amazonensis* infection is followed by dramatic increases in SOD-1 expression and activity, which has been shown to directly favor parasite survival.^27^

**Fig. 6:**
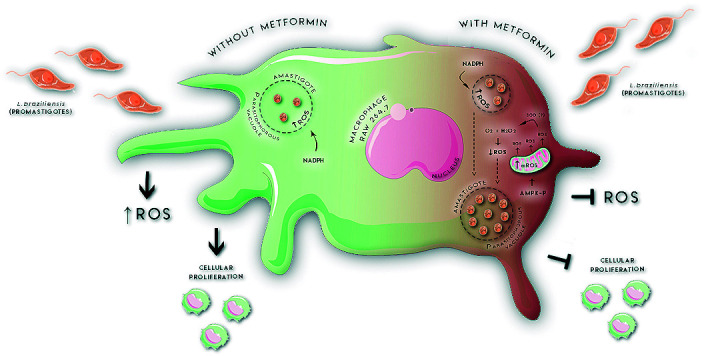
proposed model of metformin-induced immunomodulation during infection by *Leishmania braziliensis (Lb)* in macrophages. Phagocytosis triggers the production of reactive oxygen species (ROS) via NADPH oxidase inside the parasitophorous vacuole. The absence metformin (MET) also does not interfere with the cellular proliferation and ensures greater control of parasitic load due to the absence of subversion of microbicidal macrophage mechanisms. Upon the introduction of MET, activated protein kinase (AMPK)-p is activated, suppresses cell proliferation and increases mROS. Phagocytosis induces ROS via NADPH oxidase, adding to the mROS generated via AMPK-p, providing an environment with high intracellular oxidative stress. This generated condition activates the superoxide dismutase (SOD) detoxification pathway of the host cell, as well as its isoforms in the parasite, reducing intracellular ROS and enabling parasitic growth inside the parasitophorous vacuole.

The data presented herein allow us to propose (Fig. 6) that the use of MET decreases cell proliferation in *Lb* infection and blocks the release of ROS into the extracellular medium. In addition, elevated production of ROS induced by *Leishmania* and the MET pathway, through the increase of AMPK-p, can generate high levels of cellular oxidative stress and promote the activation of host SOD production and its described isoforms in parasites,^28^ which will induce the detoxification of ROS and increase parasite load inside parasitophorous vacuoles.

Our experimental findings suggest that MET, the main hypoglycemic drug used in the treatment of diabetes, does not aid in the control of *Lb* infection, and may actually exacerbate viability and parasite load.
